# Brain Swelling and Loss of Gray and White Matter Differentiation in Human Postmortem Cases by Computed Tomography

**DOI:** 10.1371/journal.pone.0143848

**Published:** 2015-11-30

**Authors:** Go Shirota, Wataru Gonoi, Masanori Ishida, Hidemi Okuma, Yukako Shintani, Hiroyuki Abe, Yutaka Takazawa, Masako Ikemura, Masashi Fukayama, Kuni Ohtomo

**Affiliations:** 1 Department of Radiology, Graduate School of Medicine, The University of Tokyo, 7-3-1 Hongo, Bunkyo-ku, Tokyo 113–8655, Japan; 2 Department of Radiology, Mutual Aid Association for Tokyo Metropolitan Teachers and Officials, Sanraku Hospital, 2–5 Kandasurugadai, Chiyoda-ku, Tokyo 101–8326, Japan; 3 Department of Pathology, Graduate School of Medicine, The University of Tokyo, 7-3-1 Hongo, Bunkyo-ku, Tokyo 113–8655, Japan; INSERM U894, FRANCE

## Abstract

The purpose of this study was to evaluate the brain by postmortem computed tomography (PMCT) versus antemortem computed tomography (AMCT) using brains from the same patients. We studied 36 nontraumatic subjects who underwent AMCT, PMCT, and pathological autopsy in our hospital between April 2009 and December 2013. PMCT was performed within 20 h after death, followed by pathological autopsy including the brain. Autopsy confirmed the absence of intracranial disorders that might be related to the cause of death or might affect measurements in our study. Width of the third ventricle, width of the central sulcus, and attenuation in gray matter (GM) and white matter (WM) from the same area of the basal ganglia, centrum semiovale, and high convexity were statistically compared between AMCT and PMCT. Both the width of the third ventricle and the central sulcus were significantly shorter in PMCT than in AMCT (*P* < 0.0001). GM attenuation increased after death at the level of the centrum semiovale and high convexity, but the differences were not statistically significant considering the differences in attenuation among the different computed tomography scanners. WM attenuation significantly increased after death at all levels (*P*<0.0001). The differences were larger than the differences in scanners. GM/WM ratio of attenuation was significantly lower by PMCT than by AMCT at all levels (*P*<0.0001). PMCT showed an increase in WM attenuation, loss of GM–WM differentiation, and brain swelling, evidenced by a decrease in the size of ventricles and sulci.

## Introduction

Postmortem computed tomography (PMCT) is a noninvasive tool that can be used to investigate cause of death. Use of computed tomography (CT) is gaining popularity in postmortem investigations as an adjunct to more traditional methods in clinical pathology and forensic medicine [1–9]. PMCT findings consist of three components: (1) changes related to the cause of death; (2) nonspecific natural changes occurring in the agonal stage and/or after death; and (3) effects of cardiopulmonary resuscitation. It is important to distinguish between these three components.

At present, the use of PMCT for postmortem investigations is still in its infancy and is a complimentary tool to conventional autopsy. Thus, findings by PMCT need to be carefully compared with conventional autopsy results. Therefore, investigations using CT just before autopsy are required in order to objectively compare results by CT and autopsy. PMCT of the brain is especially important, given that it is important to distinguish changes caused by “primary” intracranial fatal events and nonspecific changes that occur in the agonal stage or after cessation of circulation.

Diffuse brain swelling and loss of gray matter (GM)–white matter (WM) differentiation is seen on CT for a variety of “primary” fatal conditions, such as trauma [10], cerebral air embolism [11], acute necrotizing encephalopathy [12], fat-embolism [13], and so on. These changes are also seen in global central nervous system hypoperfusion or hypoxic-anoxic encephalopathy “secondary” to cardiopulmonary arrest due to any causes [14, 15] and empirically noted that they are also nonspecific common findings in PMCT [16]. If we can quantitatively prove that these findings are seen in PMCT for subjects who are pathologically proven to have no primary intracranial abnormal changes, we can consider these findings nonspecific and not pathognomonic.

One previous study quantified the loss of GM–WM differentiation by comparing GM/WM ratios of CT number between PMCT and antemortem CT (AMCT) [17]. However in this previous study, autopsies were performed only in three cases out of 41 cases enrolled in the study.

To our knowledge, studies comparing alterations in the brain by AMCT, PMCT, and autopsy of the same subject have never been reported. The aim of this study was to evaluate and compare brain PMCT and AMCT features in terms of brain swelling and loss of GM–WM differentiation in the same patients.

## Materials and Methods

### Study group

The Research Ethics Committee of The University of Tokyo Hospital approved this study, which was conducted in accordance with the principles of the Declaration of Helsinki. Written informed consent was obtained from the next of kin of the donor for all corresponding clinical and radiographic data to be used in the study. A total of 66 patients who died from nontraumatic disease in our academic tertiary-care hospital, and who underwent AMCT, PMCT, and pathological autopsy including brain evaluation between April 2009 and December 2013 were retrospectively enrolled in this study. Exclusion criteria were as follows: (1) findings of pathological autopsy and/or AMCT/PMCT revealing massive intracranial disorders that may relate to cause of death or that may affect measurement in our study, such as a massive intracranial tumor, large infarction, or intracranial hemorrhages; (2) the date of the last AMCT of the head conducted more than 24 months before the date of death; (3) injection of intravenous contrast enhancement material within 48 h before the time of death. The final study population consisted of 36 adult human cadavers (27 male, 9 female); mean age at death was 72.9 years (range, 45‒98 years; median, 74 years). All cadavers were placed in the supine position at room temperature from the time of death until PMCT examination. PMCT was performed at 90‒1151 min (median 291 min) after death.

### CT technique

All AMCT studies were performed on multi-detector CT scanners (Aquilion 64 and Aquilion ONE, Toshiba Medical Systems Corporation, Ohtawara, Japan; Discovery CT750 HD and LightSpeed VCT, GE Healthcare, Buckinghamshire, UK) with parameters recommended by the manufacturers (Table 1). All AMCT scans were done in conventional mode, except for one subject that was scanned using Aquilion ONE in the volume scan mode. All PMCT studies were performed on a 4-detector-row CT scanner (Robusto, Hitachi Medical Corporation, Tokyo, Japan) in the conventional mode with the scan parameters also shown in Table 1.

**Table 1:** Acquisition parameters for each CT scanner.

AMCT				
Scanner	current (mA)	voltage (kV)	slice thickness (mm)	number of subjects
Aquillion 64	200	120	4	16
Aquillion ONE	200	120	4	3
	270*	120	5	1
Discovery CT 750 HD	200	120	5	7
	114–199**	120	5	3
LightSpeed VCT	200	120	5	6
total				36
PMCT	current (mA)	voltage (kV)	slice thickness (mm)	number of subjects
Scanner				
ROBUSTO	200	120	5	36

*Volume Scan mode

**Tube current was controlled automatically using Smart mA®.

### Imaging analysis

PMCT scans were compared with the latest AMCT scans. The latest AMCT analyses used for comparison were performed within 12 months before death for 31 of 36 (86.1%) subjects, and 12–19 months for 5 of 36 (13.9%) subjects. Median interval between AMCT and PMCT was 43.4 days.

**Fig 1:**
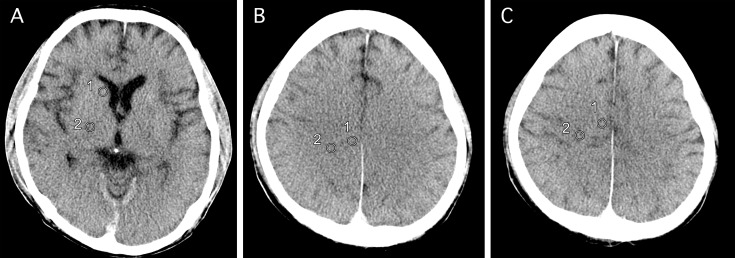
Region of interest (ROI) at each level. The three ROIs that were analyzed in our study include the basal ganglia level, defined as the image in which the caudate nucleus, internal capsule, third ventricle, and sylvian fissures were visualized (A), centrum semiovale level defined as the image one slice above the lateral ventricular system (B), and high convexity level defined as the next image above the centrum semiovale level (C). In each case, cursor 1 is on the GM and cursor 2 is on the WM. The size of each ROI is 10 mm^2^.

To test for brain swelling we measured the width (length of the minor axis) of the third ventricle at the same level of the basal ganglia, and the width of the central sulcus at the level of the high convexity. We measured attenuation (Hounsfield units, HUs) in the GM and WM that were obtained from the same area of the basal ganglia, centrum semiovale, and high convexity level in AMCT and PMCT. These three regions of interest were defined according to the method by Torbey et al. 2000 [15], which was adopted in another previous study [17], as shown in Fig 1. To quantify the loss of GM–WM differentiation, we compared GM/WM ratios of attenuation in HUs between PMCT and AMCT.

Imaging analysis was performed by a board-certified radiologist with experience in postmortem imaging (G.S.), who was not provided with clinical information.

### Imaging phantom analysis

Because we used different CT scanners for AMCT and PMCT, we scanned the same imaging phantom to verify the feasibility of comparing attenuation among machines before comparing CT attenuation between AMCT and PMCT. We scanned the head portion of the whole body imaging phantom (PBU-60, Kyoto Kagaku Co., Ltd., Kyoto, Japan) at the level of the basal ganglia, centrum semiovale, and high convexity with each scanner, except for the 4-detector-row CT scanner (LightSpeed VCT). Then we measured attenuation obtained from the same area of the basal ganglia, centrum semiovale, and high convexity.

### Autopsy

All autopsies were performed by board-certified pathologists immediately following the PMCT scans. The pathologists were informed of the patient clinical history and PMCT findings. Autopsies including brain were performed according to traditional procedures.

Presence of hyperemia of the brain surface was evaluated on the basis of the photographs of the specimens before and after formalin fixation, if available, by a neuropathologist (M.I.) without the information of the PMCT findings.

### Statistical analyses

All statistical analyses were performed using the free software R, version 2.7 (The R Foundation for Statistical Computing, Vienna, Austria, http://www.r-project.org/). The normality of each variable was tested using the Shapiro–Wilk test.

We compared the width of the third ventricle at the same level of the basal ganglia and the width of the central sulcus at the level of high convexity between AMCT and PMCT with Wilcoxon signed rank test, because normality of the variable was rejected on both AMCT and PMCT. We compared attenuation of GM and WM between AMCT and PMCT at each level. We then compared the attenuation ratio of GM to WM between AMCT and PMCT at each level. For these comparisons, we used paired t-test if the normality of variable was proven, or Wilcoxon signed rank test if the normality of variable was rejected.

In order to investigate the relationship between the time after death and the indicators of brain swelling, we conducted correlation analysis between the time after death and the ratio of PMCT to AMCT for both the width of the third ventricle and the central sulcus. We also conducted correlation analysis between the time after death and the ratio of PMCT to AMCT for CT attenuation of both GM and WM at each level.

In some previous studies, parenchymal hyperemia was considered as one of the etiologies of WM attenuation change [15, 18]. Based on the assumption that hyperemia of the brain surface reflects parenchymal hyperemia, we compared WM attenuation ratio of PMCT to AMCT between the cases in which hyperemia of the brain surface in the autopsied specimen was present and the cases in which it was not obvious.

In order to make sure that the AMCT-PMCT interval was negligible in our analysis, we also conducted Mann–Whitney U-test for the indicators of brain swelling and attenuation change between the cases whose AMCT-PMCT interval was longer than the median (43.4 days) and the cases whose AMCT-PMCT interval was shorter than the median. The level of statistical significance was set at *P*≤0.05.

## Results

### Autopsy findings

#### Cause of death

The autopsy findings of the causes of death were: respiratory failure (17 cases), multiple organ failure due to neoplasm (7 cases), sepsis (5 cases), lethal arrhythmia (1 case), cholesterol embolism (1 case), nonbacterial thrombotic endocarditis (1 case), ischemic heart disease (1 case), cardiac tamponade (1 case), multiple thromboembolism due to aortic dissection (1 case), generalized peritonitis (1 case). Two subjects had undergone CPR before death.

#### Pathological findings of the brain

Our study excluded subjects with massive intracranial disorders that may be related to the cause of death or that may affect measurements by pathological autopsy and/or PMCT. Nevertheless, the following findings were revealed: lacunar infarctions and/or multiple infarctions that may not affect our measurement (15 cases), microscopic brain infiltration of a neoplasm (4 cases), small chronic subdural hematoma (2 cases), dementia with Lewy bodies (1 case), central pontine myelinolysis (1 case), meningioma (1 case). There was no evidence of brain tissue autolysis in any of the cases.

Photographs of the brain specimens before and after formalin fixation were available in 35 cases. In 18 cases hyperemia of the brain surface was present, while in 17 cases it was not obvious.

### Imaging phantom study

The CT attenuations of imaging phantoms at each level with each machine are shown in Table 2.

**Table 2:** Imaging phantom study.

				CT attenuation (HU)		
Scanner	current (mA)	voltage (kVp)	slice thickness (mm)	BG	CS	HC
Aquillion 64	200	120	4	35	35	35
Aquillion ONE	200	120	4	34	34	35
Discovery CT 750 HD	200	120	5	35	37	35
	Smart mA*	120	5	35	38	35
ROBUSTO	200	120	5	35	36	37

BG: basal ganglia; CS: centrum semiovale; HU: Hounsfield unit; HC: high convexity

*Tube current was controlled automatically using Smart mA®.

The results suggest that HU differences among different machines are within 1 HU at the basal ganglia level, 4 HUs at the centrum semiovale level, and 2 HUs at the high convexity level.

### AMCT-PMCT comparison

#### Widths of the ventricles and the central sulci

**Fig 2:**
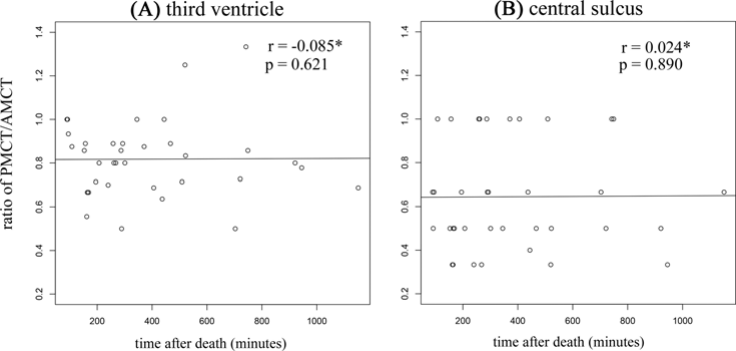
Correlation between time after death and changes in the width of the third ventricle and the central sulcus. (A) Scatter plot of the ratio of PMCT/AMCT in the width of the third ventricle and time after death. (B) Scatter plot of the ratio of PMCT/AMCT in the width of the central sulcus and time after death. *Spearman’s rank correlation.

The widths of the ventricles and the central sulci were compared between AMCT and PMCT. Both the widths of the third ventricles and the central sulci were significantly shorter in PMCT compared with AMCT (Table 3). These changes in the indicators reflect brain swelling in PMCT. Changes in the indicators above did no correlate with the time after death (Fig 2).

**Table 3:** Widths of the third ventricle and central sulcus.

	AMCT	PMCT	p value
Third ventricle (mm, mean ± SD)	9.02 ± 3.15	7.28 ± 2.69	<0.0001*
Central sulcus (mm, mean ± SD)	2.61 ± 0.93	1.56 ± 0.56	<0.0001*

SD: standard deviation

*Statistical analyses were performed by Wilcoxon signed-rank test.

There was no significant difference in change of the third ventricle size between the cases whose AMCT-PMCT interval was longer than the median (43.4 days) and the cases whose AMCT-PMCT interval was shorter than the median (Table 4). However, there was a significant difference in change in width of the central sulcus between the cases whose AMCT-PMCT interval was longer than the median and the cases whose AMCT-PMCT interval was shorter than the median. Then we separately compared the width of the central sulcus between AMCT and PMCT within a subgroup of cases with short AMCT-PMCT interval and within a subgroup of cases with long AMCT-PMCT interval (Table 5).

**Table 4:** Relationship between AMCT-PMCT interval and the brain swelling indicators.

PMCT/AMCT ratio	AMCT-PMCT interval shorter than the median	AMCT-PMCT interval longer than the median	p value*
Third ventricle (mean ± SD)	0.79 ± 0.18	0.84 ± 0.17	0.1769
Central sulcus (mean ± SD)	0.54 ± 0.20	0.75 ± 0.24	0.0132

SD: standard deviation

*Statistical analyses were performed by Mann–Whitney *U* test.

**Table 5:** Width of central sulcus changes for cases with short AMCT-PMCT interval and long AMCT-PMCT interval.

	AMCT	PMCT	p value
AMCT-PMCT interval shorter than the median (n = 18, mm, mean ± SD)	2.78 ± 0.81	1.44 ± 0.51	<0.0001*
AMCT-PMCT interval longer than the median (n = 18, mm, mean ± SD)	2.44 ± 1.04	1.67± 0.59	<0.002*

SD: standard deviation

*Statistical analyses were performed by Wilcoxon signed-rank test.

#### GM and WM attenuation

**Fig 3:**
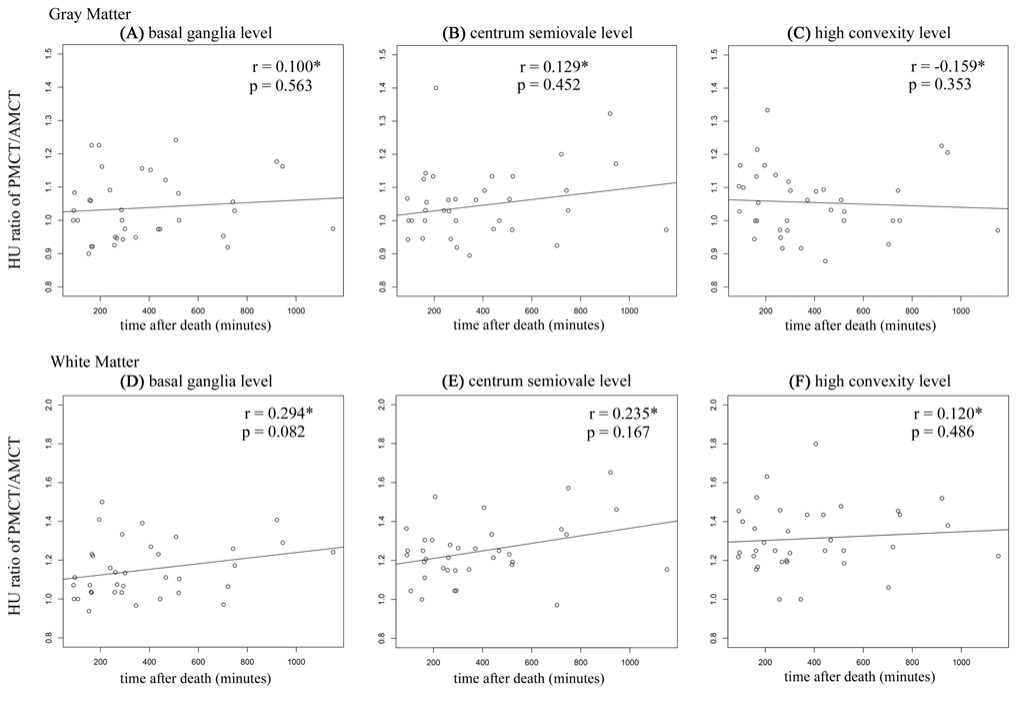
Correlation between time after death and attenuation change. Scatter plot of HU ratio of PMCT/AMCT in GM and time after death (A: basal ganglia level; B: centrum semiovale level; C high convexity level). Scatter plot of HU ratio of PMCT/AMCT in WM and time after death (D: basal ganglia level; E: centrum semiovale level; C: high convexity level). *Spearman’s rank correlation.

Attenuation of GM and WM at each level are shown in Table 6. GM attenuation significantly increased in PMCT at the level of the centrum semiovale and high convexity. However, considering the result from our imaging phantom study, these differences were relatively smaller than the differences of CT attenuations among the different scanners. WM attenuation was significantly increased after death at all levels. These differences were large enough even when taking into account the differences of CT attenuations among the different scanners. The GM/WM ratio of attenuation was significantly lower in PMCT compared with AMCT at all levels (Table 7). The change in attenuation did not correlate with the time after death with regard to GM and WM at all levels (Fig 3).

**Table 6:** Attenuation of GM and WM at various axial levels.

	AMCT	PMCT	p value
Gray matter			
Basal ganglia (HU; mean ± SD)	35.61 ± 3.21	36.72 ± 2.25	0.0518*
Centrum semiovale (HU; mean ± SD)	33.22 ± 3.20	34.44 ± 2.76	0.0314**
High convexity (HU; mean ± SD)	33.53 ± 3.38	35.11 ± 2.16	0.0057**
White matter			
Basal ganglia (HU; mean ± SD)	28.03 ± 2.91	31.94 ± 2.63	< 0.0001**
Centrum semiovale (HU; mean ± SD)	24.47 ± 3.28	30.25 ± 3.74	< 0.0001**
High convexity (HU; mean ± SD)	24.44 ± 3.49	31.66 ± 3.11	< 0.0001*

HU: Hounsfield unit; SD: standard deviation

*Statistical analyses were performed by paired t-test.

**Statistical analyses were performed by Wilcoxon signed-rank test.

**Table 7:** GM/WM ratio at various axial levels.

	AMCT	PMCT	p value
Basal ganglia (mean ± SD)	1.28 ± 0.11	1.15 ± 0.08	<0.0001*
Centrum semiovale (mean ± SD)	1.37 ± 0.18	1.15 ± 0.12	<0.0001**
High convexity (mean ± SD)	1.39 ± 0.20	1.12 ± 0.11	<0.0001**

SD: standard deviation

*Statistical analyses were performed by paired t-test.

**Statistical analyses were performed by Wilcoxon signed-rank test.

There were no significant differences in WM attenuation elevation between the cases for which hyperemia of the brain surface was present in the autopsied specimen (18/36), and in the cases in which it was not obvious (17/36) (Table 8). However at the level of the basal ganglia and high convexity, there was a tendency of attenuation change being greater in cases in which hyperemia of the brain surface was present.

**Table 8:** Attenuation change in the WM and hyperemia of the brain surface in the autopsied specimen.

PMCT/AMCT ratio of White Matter attenuation	Hyperemia (+) n = 18	Hyperemia (-) n = 17	p value*
Basal ganglia (mean ± SD)	1.16 ± 0.14	1.14 ± 0.16	0.4186
Centrum semiovale (mean ± SD)	1.26 ± 0.18	1.23 ± 0.13	0.8513
High convexity (mean ± SD)	1.34 ± 0.18	1.29 ± 0.16	0.5402

SD: standard deviation

*Statistical analyses were performed by Mann–Whitney *U* test.

There were no significant differences in the attenuation change between the cases in which the AMCT-PMCT interval was longer than the median (43.4 days) and in the cases in which the AMCT-PMCT interval was shorter than the median (Table 9).

**Table 9:** Relationship between AMCT-PMCT interval and attenuation change.

PMCT/AMCT ratio of attenuation		AMCT-PMCT interval shorter than the median	AMCT-PMCT interval longer than the median	p value*
Gray Matter				
	Basal ganglia (mean ± SD)	1.04 ± 0.10	1.04 ± 0.10	0.9813
	Centrum semiovale (mean ± SD)	1.07 ± 0.13	1.02 ± 0.11	0.8083
	High convexity (mean ± SD)	1.05 ± 0.12	1.05 ± 0.08	0.9314
White Matter				
	Basal ganglia (mean ± SD)	1.15 ± 0.16	1.15± 0.13	0.7250
	Centrum semiovale (mean ± SD)	1.24 ± 0.17	1.25 ± 0.14	0.3426
	High convexity (mean ± SD)	1.29 ± 0.16	1.34 ± 0.17	0.3927

SD: standard deviation

*Statistical analyses were performed by Mann–Whitney *U* test.

## Discussion

It has been empirically stated that brain swelling and the loss of GM–WM differentiation are seen in PMCT [3, 16]. However, there have not been many quantitative studies on these phenomena. Yen et al 2007 [3] revealed brain swelling in PMCT; however, in their study the majority of their subjects (45/57) had suffered from head trauma. Another previous study quantitatively compared AMCT and PMCT in nontraumatic cases, but in their study only three out of 41 cases were autopsied [17]. Our study is the first quantitative analysis on brain swelling and change in attenuation of PMCT for nontraumatic cases, which were all autopsied and proven to have no abnormal pathological changes in the brain that might be related to the cause of death. We revealed some differences in results between the previous studies and ours. Discussion on the reason of the discrepancy may contribute to further understanding of findings in PMCT.

One of the discrepancies that we have to explain is the existence of brain swelling in PMCT. One of the previous quantitative studies concluded that the sizes of the third ventricle and the central sulcus did not significantly differ between AMCT and PMCT [17]. However, in our study population, the widths of the third ventricle and the central sulcus were significantly shorter in PMCT compared with AMCT, which reflects brain swelling after death.

According to previous studies, the pathology of brain swelling in PMCT can be explained mainly by two factors. The first factor is vasogenic edema and hyperemia during the agonal stage [19], and the second factor for brain swelling is autolysis, which starts to be obvious 24 h after death [16]. We can rule out the latter factor, because in our study PMCT was performed within 20 h after death and there was no evidence of autolysis in a following autopsy. As shown on Fig 2, there was no correlation between the time after death and the indicators of brain swelling. From this result, we suggest the hypothesis that brain swelling occurs during the agonal stage and does not progress at least before 24 h after death when the changes by autolysis start to become obvious.

Our hypothesis is that in our study population, the brain swelling in PMCT was caused by vasogenic edema and hyperemia in the agonal stage. In cases of non-sudden deaths, there is an agonal stage of certain length during which hypoxic reperfusion of the brain occurs. On the contrary, in sudden death, hyperemia and associated brain swelling are unlikely to occur, as there is neither venous blood drainage nor cerebral blood supply after cessation of circulation. An explanation for the discrepancy in results between our study and the previous study that concluded the absence of brain swelling in PMCT [17] is the differences in the cause of death in the study populations. In our study, the most frequent cause of death was respiratory failure (17 of 36 cases). The rate of sudden death was low in our study. On the contrary, in the previous study, most PMCTs were conducted in the emergency department, and the most frequent cause of death in the previous study was cardiac sudden death (14 of 41 cases). Their subjects also included four cases of sudden deaths due to unknown origins, and four cases of thoracic aortic ruptures due to acute aortic dissections (in three cases) and aortic aneurysm (in one case). The cause of death can result in differences in the lengths of the hypoxic period in the agonal stage. Hypoxic circulation results in a compensatory dilation of intracranial vessels and can cause brain swelling.

In our study population, GM/WM ratio of attenuation was significantly lower by PMCT than by AMCT at all levels. The loss of differentiation between GM and WM in our study was mainly due to an increase in WM attenuation. The increased attenuation in PMCT was relatively more drastic in the WM than in the GM, and this contributed to loss of GM–WM differentiation in PMCT. In a previous study on the change of attenuation of GM and WM in early postmortem CT in subjects after CPR, there was increased attenuation in WM compared with normal control [18]. The authors attribute this change to the same mechanism described in another preceding study on the loss of differentiation between GM and WM in comatose patients after cardiac arrest [15]. They explain this result by suggesting that an increase in intracranial pressure can partially occlude the subependymal veins and impede deep venous outflow. Initially, the cerebral blood vessels collapse so as to decrease intracranial volume and prevent further increases in intracranial pressure. However, if systemic hypotension is corrected, as happens in the acute management of cardiac arrest, cerebral blood flow increases, resulting in the deep medullary veins becoming distended. This results in a situation in which WM becomes distended with blood and appears denser in CT scans. Subjects in this previous study are patients resuscitated after cardiac arrest; therefore it is different from our study. However, in the agonal stage, if the intracranial circulation is maintained to some extent even without CPR, a similar phenomenon can occur and lead to increased attenuation.

In our study, we compared WM attenuation ratio of PMCT to AMCT between the cases in which hyperemia of the brain surface in the autopsied specimen was present and in the cases in which it was not obvious. Although there were no significant differences, there were tendencies where attenuation change was greater in the cases in which hyperemia of the brain specimen was present. This finding is compatible with our assumption that attenuation changes in PMCT may reflect hyperemia. In addition, PMCT presumably can detect subtle parenchymal hyperemia that is not obvious in macroscopic observation.

While conducting correlation analyses between HU ratios of PMCT/AMCT and the time after death for both GM and WM at each level, we found that the change in attenuation did not correlate with the time after death at all levels. From this result we presume that once attenuation has changed during the agonal period or after circulation is arrested, it does not change for a certain period of time in the early postmortem stage. However, for investigating the time-related course of CT findings after death, the scanning of identical cadavers several times over a postmortem period is necessary for future studies. If for ethical reasons this type of investigation using human cadavers is difficult, one should consider using animal models instead, which has already been performed to study the time-related course of postmortem lung changes [20].

The time interval between the latest AMCT used as control and PMCT varied in our study population. AMCTs were performed within 12 months before death for 31 of 36 (86.1%) subjects, and 12–19 months for 5 of 36 (13.9%) subjects. As shown in Table 4 and Table 9, there were no significant differences in change in ventricle size and the attenuation changes between the cases with short AMCT-PMCT interval and the cases with long AMCT-PMCT interval. We can presume that the AMCT-PMCT interval does not have a major influence on our analysis. For the changes in width of the central sulcus, there was a significant difference between the cases with short AMCT-PMCT interval and the cases with long AMCT-PMCT interval. However, as shown in Table 5, we separately compared the width of the central sulcus between AMCT and PMCT within a subgroup of cases with a short AMCT-PMCT interval and within a subgroup of cases with a long AMCT-PMCT interval, and we found that the width of the central sulcus was significantly shorter in PMCT than in AMCT regardless of AMCT-PMCT interval.

One of the greatest limitations of our study is that the examinations were performed using different CT scanners for AMCT and PMCT, as well as different protocols. Interscanner variability of CT attenuation values is an issue that needs to be considered [21–23]. A previous study revealed that CT attenuation values are independent of tube current [24]. Another experimental study showed that they are dependent on tube voltage. The authors have also shown that CT attenuation differences between scanners are smaller at 120 kV compared with 80 kV [25]. In the present study, tube voltage was set to 120 kV for all scanners, but tube current was variable. These facts may support the results from our imaging phantom analysis, where we showed very little CT attenuation differences between the scanners. We conducted the study with the assumption that it was possible to compare attenuation between AMCT and PMCT in at least the WM.

Our study has another limitation. We did not consider pre-existing systemic disorders or antemortem blood markers that can potentially affect PMCT findings [7]. An additional investigation with more cases should be considered to reveal the relationship between antemortem hematological markers and PMCT findings.

## Conclusions

This is the first quantitative analysis that shows brain swelling and change in attenuation in PMCT compared with AMCT for in-hospital nontraumatic deaths that were not due to intracranial causes as determined by conventional autopsy. At the postmortem stage when pathological autopsies are conducted, PMCT showed an increase in WM attenuation, loss of GM–WM differentiation, and brain swelling represented by decreases in the sizes of the ventricles and sulci. These changes in the CT of the brain are nonspecific and not pathognomonic.
